# A Pilot Study on the Feasibility and Integration of High-Risk Human Papillomavirus (HPV) Testing for Cervical Cancer Prevention in Trinidad and Tobago

**DOI:** 10.7759/cureus.78389

**Published:** 2025-02-02

**Authors:** Vishal Bahall, Adesh Sirjusingh, Sally Ishmael, Yasmin Hosein, Moira Lindsay, Mickhaiel Barrow, Lance De Barry

**Affiliations:** 1 Obstetrics and Gynaecology, The University of the West Indies, St. Augustine, TTO; 2 Obstetrics and Gynaecology, San Fernando General Hospital, San Fernando, TTO; 3 Obstetrics and Gynaecology, Ministry of Health, Port-of-Spain, TTO; 4 Obstetrics and Gynaecology, North West Regional Health Authority, Port-of-Spain, TTO; 5 Obstetrics and Gynaecology, Port-of-Spain General Hospital, Port-of-Spain, TTO; 6 Sociology, Population Services International (PSI) Caribbean, Port-of-Spain, TTO; 7 Pathology, Port of Spain General Hospital, Port-of-Spain, TTO

**Keywords:** cervical cancer prevention, cervical cancer screening, hpv testing, human papillomavirus (hpv), pap smear test

## Abstract

Background

Globally, cervical cancer is one of the leading causes of cancer-related death among women. Persistent infection with high-risk human papillomavirus (hrHPV) is the predominant etiological factor for cervical cancer, underscoring the critical need for effective screening to enable early detection and reduce disease burden. However, in Trinidad and Tobago, liquid-based cytology is not available in the public healthcare system, and current screening efforts rely solely on conventional Pap smears. This limitation restricts the integration of hrHPV testing either as a standalone primary screening tool or as part of a co-testing strategy. Addressing these gaps through infrastructure investment and policy reform is essential for achieving comprehensive and equitable cervical cancer prevention.

Objectives

This study aimed to evaluate the feasibility of hrHPV testing within the public healthcare system of Trinidad and Tobago, identifying potential facilitators and barriers to nationwide implementation.

Methods

A descriptive cross-sectional study design was employed. Women aged 30-65 meeting specific inclusion criteria were recruited from 18 primary healthcare centers and a mobile unit between March 2019 and November 2019. HPV samples were collected, analyzed using the careHPV system, and categorized as positive or negative for high-risk HPV types. Patients with positive results underwent follow-up conventional cytology, with colposcopy referral as indicated by cytological findings.

Results

Out of 1241 women tested, 1201 were eligible. hrHPV positivity was detected in 140 women (11.7%), with the highest positivity rate observed in 75 women (15.7%) out of a cohort of 476 women in their 30s. Of those with positive results, 115 women (81.6%) were referred for cytology, revealing 12 cases of high-grade squamous intraepithelial lesions and a CIN 2/3 incidence of 46.7% (14 cases) among 30 women with concurrent positive HPV and abnormal cytology. Active call-and-recall mechanisms led to increased screening uptake by 33% within the target catchment area.

Conclusion

hrHPV testing demonstrated practical feasibility and improved sensitivity for detecting pre-invasive cervical lesions compared to cytology alone. Implementing a screening algorithm integrating HPV and cytology testing could optimize patient triage, alleviate colposcopy service demand, and enhance screening coverage. When combined with effective HPV vaccination campaigns, these measures present a viable path toward achieving the World Health Organization's cervical cancer elimination goals in Trinidad and Tobago.

## Introduction

Globally, cervical cancer is the fourth most common cancer in women [1]. Approximately 570,000 women were diagnosed with cervical cancer worldwide in 2018 [2]. Of the 311,000 deaths that year attributed to cervical cancer, up to 90% occurred in low and middle-income countries [2].

In Trinidad and Tobago, cervical cancer ranks as the second most prevalent malignancy and continues to be a leading contributor to cancer-related mortality in women [3]. Annually, approximately 140 new cases are diagnosed, and an estimated 97 women succumb to the disease [4]. The impact of cervical cancer is especially pronounced in developing countries, largely attributed to factors such as insufficient public awareness, limited access to healthcare services, and the absence of a structured and effective screening program [5].

Persistent human papillomavirus (HPV) infection is the most important contributing factor in the development of cervical cancer [6]. HPV is composed of a group of viruses acquired sexually. HPV subtypes are often referred to as “high-risk” or “low-risk” based on their oncogenic potential [7]. There are approximately 13 oncogenic HPV strains, and these subtypes include 16, 18, and 45, among others [7]. On the contrary, the “low-risk” HPV subtypes, such as 6 and 11, cause genital warts and do not predispose to cervical cancer [7]. In the majority of women, HPV viral clearance typically occurs within a few months, and up to 90% of cases are undetectable within two years [8]. However, HPV infections can persist and lead to pre-cancerous lesions within the cervix [8]. If left undiagnosed and untreated, these pre-cancerous lesions can progress to invasive cervical cancer, often taking 15 to 20 years to develop in women with normal immune function [8]. This extended window presents a critical opportunity for effective screening and prevention strategies.

Cervical cancer screening serves as a pivotal form of secondary prevention designed to identify and manage abnormal cervical cells at an early stage, thereby reducing the risk of progression to invasive disease. The World Health Organization (WHO) recommends three screening modalities to enhance detection rates. These include high-risk HPV (hrHPV) DNA testing, which is highly sensitive for detecting high-risk HPV subtypes known to be associated with cervical cancer [9]. Visual inspection with acetic acid (VIA) offers a cost-effective and immediate screening option that is particularly beneficial in low-resource settings [9]. The Pap smear, one of the oldest and most established screening tools, remains widely used due to its ability to detect cellular changes, while liquid-based cytology offers a refined method with improved sample preservation and diagnostic accuracy [9].

Several factors promote hrHPV testing as the primary screening tool. According to epidemiological studies, hrHPV testing demonstrates superior sensitivity and effectiveness compared to traditional Pap smear and liquid-based cytology, with an estimated 20-30% greater sensitivity for detecting high-grade cervical lesions [10]. In contrast, Pap smears are subject to variability in reproducibility and may produce false positive or false negative results, thereby limiting their reliability. The hrHPV test, however, offers a more objective approach by detecting the presence of HPV DNA and specific genotypes within cervical cell samples [11]. As a result, hrHPV testing permits an extended screening interval of five years, compared to the three-year interval required for cervical cytology, without increasing the patient's lifetime risk of developing cervical cancer [11]. Finally, hrHPV test samples can be self-collected by women, offering a less invasive and more accessible approach [12]. This method has the potential to significantly enhance public participation in screening programs due to its added convenience and ease of use [12].

Objectives

The objective of this pilot study is to assess the feasibility of hrHPV testing as a screening tool in the public healthcare system in Trinidad and Tobago. Its use in this small-scale project will aid in identifying the triumphs and barriers to hrHPV testing so that we can perform future national implementation.

## Materials and methods

The research was conducted using a descriptive cross-sectional study and approved by the Ministry of Health, Trinidad and Tobago, on 18th June 2018 (reference He:10/21/251 Vol 1). Through convenience sampling, women who met the eligibility criteria were recruited from the 18 primary healthcare centers and one mobile unit between March 2019 and November 2019. The Ministry of Health (MOH) of Trinidad and Tobago, along with the medico-legal authority within the MOH, accepted the screening protocol design with input from local and independent specialists from MD Anderson University. Trained personnel tasked with taking samples at the screening sites. Written informed consent was obtained from all patients after counseling on the study protocol.

Inclusion and exclusion criteria

The inclusion criteria were women aged 30 to 65, women screened for the first time (no previous pap smears), and women who did not undergo treatment or follow-up for an earlier abnormal smear within the last six months.

The exclusion criteria included pregnant women, women who were undergoing treatment for an abnormal smear, women who have not been referred to routine screening after an abnormal smear, women who are known to be infected with other sexually transmitted infections, and women who have undergone a total hysterectomy.

Sample size and training

One thousand five hundred (1500) hrHPV testing kits were procured to test eligible women. Staff training was conducted before patient recruitment. Four laboratory technicians and one doctor completed three weeks of laboratory training. Physicians responsible for performing HPV and cytology tests received appropriate training before recruitment. Physicians and nurses within the primary care health centers and occupational health departments were sensitized to the project. The algorithm was provided in both soft and hard copies.

Women who fulfilled the inclusion criteria were informed about the program. Interested patients consented, and a cervical sample was collected in the gynecological exam room.

Sample collection and storage

The physician took samples from the cervix using the careHPV collection brush. The samples were stored in the medium tube of the careHPV collection, which was labeled and placed in the collection area.

Samples were stored at room temperature (15 ^0^C to 30 ^0^C) and did not require refrigeration. HPV samples were collected weekly and transported to the Port-of-Spain General Hospital laboratory, where they were stored in a designated refrigerator at 4 ^0^C. At this temperature, samples can be stored for up to four weeks.

Laboratory analysis

All HPV samples were analyzed using the careHPV testing machine (Qiagen N.V., Hilden, Germany), which utilizes Hybrid Capture 2 technology. It detects the presence of HPV subtypes 16, 18, 31, 33, 35, 39, 45, 51, 56, 58, 59, 66, and 68. The results are either positive or negative.

Samples were run in batches, with a maximum of 90 samples per batch. To maximize the use of testing samples, technicians were advised to wait until they could run an entire batch of 90 samples.

Review of results

Results were reviewed weekly. Patients received their results in approximately 30 days or less in person at the health center where the test was performed or via telephone for the mobile unit. In the event of a positive result, the health center arranged a follow-up Pap smear appointment. The Pap smears were also analyzed at the Port-of-Spain General Hospital Laboratory, and abnormal results were directed to the Colposcopy Department with the patient’s contact information. The Colposcopy Department scheduled appointments directly.

Statistical analysis methods

Descriptive statistical methods were employed to analyze the data in this study. Frequencies and percentages were calculated to summarize the prevalence of hrHPV positivity and its distribution across different age groups. Proportional analysis was conducted to determine the rates of cytological abnormalities, colposcopy referrals, and histopathological outcomes among hrHPV women. Lastly, simple arithmetic comparisons were utilized to identify trends in age-specific hrHPV positivity rates and progression to high-grade cervical lesions.

Testing algorithm

**Figure 1:**
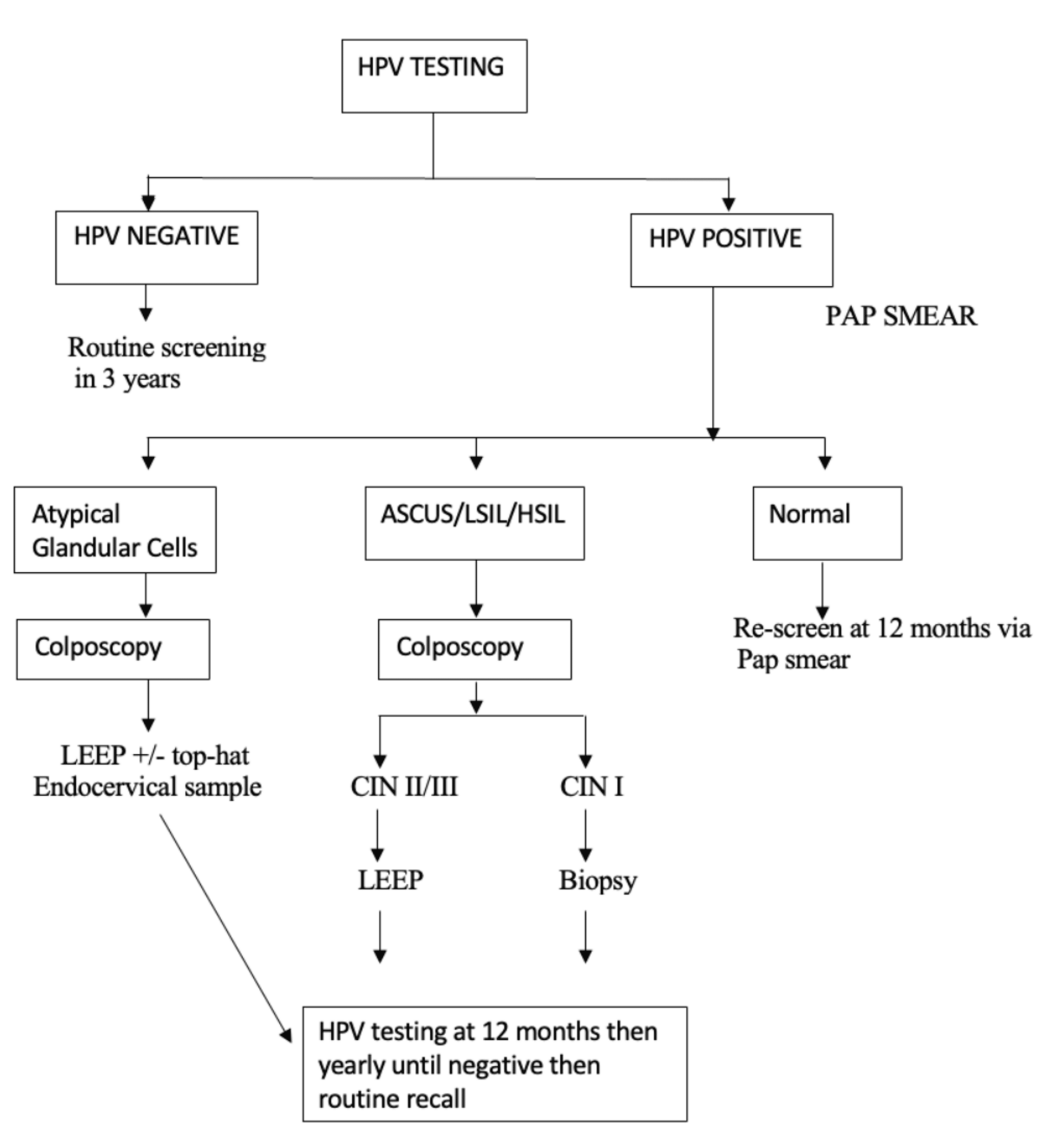
hrHPV testing algorithm. ASCUS: Atypical Squamous Cells of Undetermined Significance; LSIL: Low-Grade Squamous Intraepithelial Lesion; HSIL: High-Grade Squamous Intraepithelial Lesion; LEEP: Loop Electrosurgical Excision Procedure; hrHPV: High-Risk Human Papillomavirus

Figure 1 illustrates the testing algorithm developed per international guidelines. This algorithm was reviewed comprehensively by key stakeholders, including local and international colposcopies.

For patients with a negative hrHPV test result, a return to routine screening after three years is recommended. Conversely, a Pap smear is performed if the hrHPV test yields a positive result. In cases where the Pap smear is normal, a follow-up Pap smear is scheduled for 12 months.

A referral for colposcopy is indicated if the Pap smear demonstrates atypical squamous cells of undetermined significance (ASCUS), low-grade squamous intraepithelial lesions (LSIL), or high-grade squamous intraepithelial lesions (HSIL). During colposcopy, if cervical intraepithelial neoplasia grade 1 (CIN-I) is identified, a cervical biopsy is conducted. Detection of CIN grades II or III necessitates a loop electrosurgical excision procedure (LEEP).

For patients with abnormal Pap smears characterized by atypical glandular cells, colposcopy is recommended. This procedure could be followed by LEEP and/or a top-hat procedure, along with endocervical sampling.

All patients with abnormal Pap smear results undergo repeat hrHPV testing at 12 months, with annual retesting until a negative result is achieved, at which point they are transitioned to routine recall. Decisions regarding further excisional procedures, endometrial biopsies, or hysterectomies are made on an individualized basis, guided by the clinical judgment of the gynecologist.

## Results

A cohort of 1306 women underwent testing, with 1201 meeting the study's eligibility criteria. Among this cohort, 140 women (11.7%) tested positive for high-risk HPV. Analysis of age-specific distribution demonstrated the highest positivity rate (31.7%) among women in their 30s (Table 1).

**Table 1:** Distribution of women based on their age and positive result for “high-risk” HPV subtypes. HPV: Human Papillomavirus

Age Group in years	Number of women tested for HPV	Number of tested women included in the study	Number of women testing positive for high-risk HPV subtypes	Percentage of HPV-positive women (%)
30 - 34	286	271	41	15.1
35 - 39	211	205	34	16.6
40 - 44	184	179	17	9.2
45 - 49	151	148	13	8.6
50 - 54	142	136	16	6.3
55 - 59	152	149	9	5.9
60 - 64	104	102	9	8.7
Over 65	11	11	1	9.1
Total	1241	1201	140	11.7

Following the detection of high-risk HPV, affected individuals were prompted for conventional cytology testing. Among these, 115 women (81.6%) were referred for a conventional smear, whereas 25 women (17.9%) did not receive referrals due to specific reasons detailed in Table 2. Abnormal cytological findings led to the referral of 41 women for colposcopy; however, 11 individuals did not attend their scheduled appointments (Table 3). The results indicated 12 cases of HSIL, four cases of LSIL, and 10 instances of ASCUS. Histopathological examination of colposcopy samples confirmed CIN II-III in 14 of the 30 women who underwent further evaluation (Table 4).

**Table 2:** Reasons identified for lack of smear referral in women testing positive for high-risk HPV subtypes and contacted to have conventional smears. HPV: Human Papillomavirus

Reasons for lack of Pap smear referral	N (%)
Unable to contact or verify information	14 (53.8%)
Refused smear against medical advice	1 (3.8%)
Co-tested	3 (11.3%)
Referred directly to colposcopy	4 (15.3%)
Pap smear done privately	3 (11.5%)

**Table 3:** Number of women referred to and engaging the Colposcopy service due to abnormal smear results following HPV testing. HPV: Human Papillomavirus

Total number of women referred to Colposcopy with abnormal smears	N (%)
Number of women who had Colposcopy completed	30 (73.2%)
Number of women who did not attend their Colposcopy appointment	11 (26.8%)

**Table 4:** Distribution of histological findings on Colposcopy for women testing positive for high-risk HPV subtypes and an abnormal Pap smear. Thirty out of 41 patients referred as a result of an abnormal smear and positive high-risk HPV test attended the Colposcopy service. ^a^HSIL: High-Grade Squamous Intraepithelial Lesion; ^b^LSIL: Low-Grade Squamous Intraepithelial Lesion; ^c^ASCUS: Atypical Squamous Cells of Undetermined Significance; ^d^ASCUS-H: Atypical Squamous Cells of Undetermined Significance, cannot rule out HSIL; ^e^AGC: Atypical Glandular Cells HPV: Human Papillomavirus

		Histology based on Colposcopy, N (%)			
Smear result	Number of women referred for Colposcopy (%)	CIN-1	CIN-2	CIN-3	HPV
HSIL^a^	12 (29.3%)	1 (11.1%)	-	5 (38.4%)	1 (14.3%)
LSIL^b^	4 (9.8%)	1 (11.1%)	1 (100%)	1 (7.7%)	-
ASCUS^c^	10 (24.4%)	4 (44.4%)	-	2 (15.4%)	3 (42.9%)
ASCUS-H^d^	1 (2.4%)	1 (11.1%)	-	-	-
AGC^e^	4 (9.8%)	-	-	4 (30.8%)	-
Invasive	1 (2.4%)	-	-	1 (7.7%)	-
Reactive change	6 (14.6%)	2 (22.2%)	-	-	2 (28.6%)
Unsatisfactory	3 (7.3%)	-	-	-	1 (14.3%)
Total	41	9	1	13	7

The data underscored that 12 (29.3%) patients with an initial positive hrHPV test subsequently developed HSIL on cytological assessment. Additionally, the incidence of CIN II/III among women with both a positive HPV result and abnormal cytology was observed to be 46.7% (14 women), highlighting the clinical significance and potential for hrHPV testing to identify high-risk individuals at earlier stages of cervical disease progression.

## Discussion

Cervical cancer screening through cervical cytology was introduced in 1928, and its efficacy was demonstrated in 1941 [13]. As a direct result, the incidence of cervical cancer decreased significantly from 9.8 cases per 100,000 women in the 1970s to 4.9 cases per 100,000 women in 2009 [14]. HPV vaccination in both developed and developing nations is the next crucial step to eradicating cervical cancer within the next century. An integrated approach that combines a robust cervical screening program with HPV vaccination is particularly vital in regions where the incidence of cervical cancer exceeds 25 cases per 100,000, ensuring comprehensive prevention and early detection [15].

In Trinidad and Tobago, the cervical cancer incidence and mortality rate are 17.6 per 100,000 and 9.2 per 100,000 respectively [16]. Comprehensive analyses of cancer incidence and mortality are currently lacking, contributing to the underreporting of cervical cancer cases and the prevalence of HPV infection. The WHO defines cervical cancer elimination as achieving fewer than four cases per 100,000 [17]. To achieve the target of cervical cancer elimination within the next 30 years, Trinidad and Tobago must prioritize the implementation of high-performance screening strategies, such as hrHPV testing, in conjunction with an effective HPV vaccination initiative targeting 70% and 90% population coverage, respectively.

Efforts to introduce hrHPV testing as a primary screening modality in low-to-middle-income countries have encountered significant challenges, primarily due to resource limitations and insufficient healthcare infrastructure [18]. In Trinidad and Tobago, liquid-based cytology is unavailable in the public healthcare system, and current screening efforts rely solely on conventional Pap smears. This limitation prevents the integration of hrHPV testing either as a standalone primary screening tool or as part of a co-testing strategy. Addressing these gaps through infrastructure investment and policy reform is crucial for achieving comprehensive and equitable cervical cancer prevention.

Our study set out to determine the feasibility of implementing hrHPV testing with the current infrastructure in the public sector. hrHPV testing in our research was done using the careHPV machine, which tests for 14 high-risk subtypes. Trinidad and Tobago intends to implement high-risk hrHPV testing using the GeneXpert HPV system, which identifies HPV-16, 18, 45, and 11 other high-risk subtypes [19]. The Ministry of Health procured this system for use in all facilities over the careHPV machine used in this study. The added advantage of a system that identifies HPV-16 and 18 is that it allows direct triage to colposcopy without the need for cervical cytology for patients who are HPV-16 and 18 positive [20]. HPV-16 and 18 positivity has a sensitivity of 90.8% and a specificity of 42.6% for CIN II [19]. This system is approved for primary hrHPV testing without concurrent cytology.

Our study identified an HPV positivity rate of 11.7%, aligning closely with the 10.6% positivity rate reported by Kotaniemi-Talonen et al. [21]. We demonstrated that 29.3% of individuals with a positive hrHPV test subsequently exhibited HSIL on cervical smear evaluation. Furthermore, the incidence of CIN II among women with both a positive hrHPV test and abnormal smear findings was 46.7%, comparable to findings from a randomized controlled trial (RCT) by Naucler et al. [22].

The implementation of hrHPV testing within screening programs offers significant benefits for detecting moderate to severe pre-invasive lesions. Multiple RCTs have reported a 30% improvement in sensitivity for identifying CIN II positive lesions, predominantly associated with HPV-16 [23]. Moreover, subsequent screenings have demonstrated a 50% reduction in CIN III positive lesions. An aggregated analysis of four RCTs confirmed that hrHPV testing outperforms cytology alone in reducing cervical cancer incidence [24]. Importantly, hrHPV testing allows for extended screening intervals; individuals testing negative for HPV at initial screening can safely follow a five-year interval compared to the three-year interval recommended for conventional cytology [25].

Additionally, our findings indicate that active screening efforts can enhance patient coverage, as evidenced by a 33% increase in screened individuals within the targeted catchment area. Incorporating an active call-and-recall system is essential for achieving the World Health Organization's target of screening over 70% of the population, thereby enhancing cervical cancer prevention efforts.

We propose an integrated algorithm where hrHPV testing and conventional cytology are conducted during the same clinical visit. This streamlined approach prioritizes patients who test positive for HPV-16 or HPV-18 for immediate colposcopy evaluation (Figure 1). Additionally, individuals with other high-risk HPV subtypes and those with ASCUS or greater on cytology are also referred for colposcopy. For women with positive HPV results but normal cytology, a follow-up hrHPV test is recommended after one year. This strategy aims to optimize colposcopy service utilization without increasing the risk of undiagnosed precancerous or cancerous lesions.

Evidence provided by Gultekin et al. supports the efficacy of primary HPV screening with conventional cytology as a triage mechanism in a single visit [26]. Combining HPV and cytology screenings in this manner can address follow-up challenges, which affect approximately 18.6% of HPV-positive women, and mitigate disruptions caused by factors such as the COVID-19 pandemic [27].

Achieving sustainable success in cervical cancer prevention necessitates the establishment of a dedicated multidisciplinary team supported by a robust management information system to minimize patient attrition, streamline follow-up, and ensure care continuity [28]. This team would coordinate screening services, manage patient engagement, and implement evidence-based interventions to bridge gaps in care and enhance adherence [29]. Additionally, integrating hrHPV testing with a comprehensive HPV vaccination program is critical to reducing cervical cancer incidence and mortality, particularly in resource-limited settings like Trinidad and Tobago [29]. Given the constraints of conventional screening methods and the absence of liquid-based cytology in public healthcare, a dual focus on hrHPV testing and vaccination maximizes both early detection and primary prevention. Aligning these efforts with WHO’s targets offers a transformative pathway to reduce disease burden, promote equitable care, and ultimately achieve cervical cancer elimination.

Limitations

This pilot study presents certain limitations that may influence the generalizability of its findings. The use of convenience sampling, while pragmatic, likely introduced selection bias and potentially skewed the representation of the broader population of women aged 30-65 in Trinidad and Tobago. The sample size, though sufficient for initial feasibility assessment, constrained the statistical power for subgroup analyses and may underrepresent specific demographic or clinical subgroups.

Furthermore, the geographic scope, confined to 18 primary healthcare centers and a single mobile unit, may have excluded women from underserved or remote regions, thereby limiting inclusivity and equity in the screening cohort. Further investigations should address these limitations by employing more representative larger cohort sizes and longitudinal follow-up to establish a robust framework for cervical cancer screening in resource-constrained settings.

## Conclusions

In summary, the findings demonstrate that hrHPV testing represents a viable, effective, and sustainable alternative to conventional cytology for cervical cancer screening. HPV-based screening offers superior sensitivity for the detection of moderate to severe pre-invasive cervical lesions, thereby contributing to a reduction in cervical cancer incidence compared to cytological methods alone. The potential for extended screening intervals of five years following a negative HPV result, combined with the option for patient self-sampling, is anticipated to enhance patient engagement with screening services. Additionally, the implementation of a targeted screening algorithm can alleviate the workload on colposcopy services by prioritizing patients with high-risk HPV strains, thereby supporting the achievement of the WHO’s target of screening at least 70% of the population. When integrated with an effective HPV vaccination program, these strategies offer a promising pathway for advancing the detection and control of cervical cancer in Trinidad and Tobago.

## References

[1] Burmeister CA, Khan SF, Schäfer G, Mbatani N, Adams T, Moodley J, Prince S (2022). Cervical cancer therapies: current challenges and future perspectives. Tumour Virus Res.

[2] Arbyn M, Weiderpass E, Bruni L, de Sanjosé S, Saraiya M, Ferlay J, Bray F (2020). Estimates of incidence and mortality of cervical cancer in 2018: a worldwide analysis. Lancet Glob Health.

[3] Volaric A, Pierre C, Zadeh S (2019). The pap party: Implementation of cervical cancer awareness and screening campaign in Trinidad and Tobago. J Am Soc Cytopathol.

[4] Scott-Williams J, Hosein A, Akpaka P, Adidam Venkata CR (2023). Epidemiology of cervical cancer in the Caribbean. Cureus.

[5] Adedimeji A, Ajeh R, Pierz A (2021). Challenges and opportunities associated with cervical cancer screening programs in a low income, high HIV prevalence context. BMC Womens Health.

[6] Koshiol J, Lindsay L, Pimenta JM, Poole C, Jenkins D, Smith JS (2008). Persistent human papillomavirus infection and cervical neoplasia: a systematic review and meta-analysis. Am J Epidemiol.

[7] Burd EM (2003). Human papillomavirus and cervical cancer. Clin Microbiol Rev.

[8] Huber J, Mueller A, Sailer M, Regidor PA (2021). Human papillomavirus persistence or clearance after infection in reproductive age. What is the status? Review of the literature and new data of a vaginal gel containing silicate dioxide, citric acid, and selenite. Womens Health (Lond).

[9] (2021). WHO Guideline for Screening and Treatment of Cervical Pre-cancer Lesions for Cervical Cancer Prevention. https://www.who.int/publications/i/item/9789240030824.

[10] Ramírez AT, Valls J, Baena A (2023). Performance of cervical cytology and HPV testing for primary cervical cancer screening in Latin America: an analysis within the ESTAMPA study. Lancet Reg Health Am.

[11] Feldstein O, Gali-Zamir H, Schejter E, Feinberg T, Yehuda-Shnaidman E, Bornstein J, Levy T (2023). High-risk HPV testing vs liquid-based cytology for cervical cancer screening among 25- to 30-year-old women: a historical cohort study. Acta Obstet Gynecol Scand.

[12] Costa S, Verberckmoes B, Castle PE, Arbyn M (2023). Offering HPV self-sampling kits: an updated meta-analysis of the effectiveness of strategies to increase participation in cervical cancer screening. Br J Cancer.

[13] Vilos GA (1998). The history of the Papanicolaou smear and the odyssey of George and Andromache Papanicolaou. Obstet Gynecol.

[14] Yang DX, Soulos PR, Davis B, Gross CP, Yu JB (2018). Impact of widespread cervical cancer screening: number of cancers prevented and changes in race-specific incidence. Am J Clin Oncol.

[15] Brisson M, Kim JJ, Canfell K (2020). Impact of HPV vaccination and cervical screening on cervical cancer elimination: a comparative modelling analysis in 78 low-income and lower-middle-income countries. Lancet.

[16] Llanos AA, Warner WA, Luciani S (2017). Gynecologic cancer mortality in Trinidad and Tobago and comparisons of mortality-to-incidence rate ratios across global regions. Cancer Causes Control.

[17] Canfell K (2019). Towards the global elimination of cervical cancer. Papillomavirus Res.

[18] Casas CP, Albuquerque RC, Loureiro RB, Gollner AM, Freitas MG, Duque GP, Viscondi JY (2022). Cervical cancer screening in low- and middle-income countries: a systematic review of economic evaluation studies. Clinics (Sao Paulo).

[19] Einstein MH, Smith KM, Davis TE (2014). Clinical evaluation of the cartridge-based GeneXpert human papillomavirus assay in women referred for colposcopy. J Clin Microbiol.

[20] Wentzensen N, Schiffman M, Palmer T, Arbyn M (2016). Triage of HPV positive women in cervical cancer screening. J Clin Virol.

[21] Kotaniemi-Talonen L, Nieminen P, Anttila A, Hakama M (2005). Routine cervical screening with primary HPV testing and cytology triage protocol in a randomised setting. Br J Cancer.

[22] Naucler P, Ryd W, Törnberg S (2007). Human papillomavirus and Papanicolaou tests to screen for cervical cancer. N Engl J Med.

[23] Rijkaart DC, Berkhof J, Rozendaal L (2012). Human papillomavirus testing for the detection of high-grade cervical intraepithelial neoplasia and cancer: final results of the POBASCAM randomised controlled trial. Lancet Oncol.

[24] Ronco G, Giorgi-Rossi P, Carozzi F (2010). Efficacy of human papillomavirus testing for the detection of invasive cervical cancers and cervical intraepithelial neoplasia: a randomised controlled trial. Lancet Oncol.

[25] Rayner M, Welp A, Stoler MH, Cantrell LA (2023). Cervical cancer screening recommendations: now and for the future. Healthcare (Basel).

[26] Gultekin M, Ramirez PT, Broutet N, Hutubessy R (2020). World Health Organization call for action to eliminate cervical cancer globally. Int J Gynecol Cancer.

[27] Vassilakos P, Wisniak A, Catarino R (2021). A cross-sectional study exploring triage of human papillomavirus (HPV)-positive women by visual assessment, manual and computer-interpreted cytology, and HPV-16/18-45 genotyping in Cameroon. Int J Gynecol Cancer.

[28] Bharel M, Santiago ER, Forgione SN, León CK, Weinreb L (2015). Eliminating health disparities: innovative methods to improve cervical cancer screening in a medically underserved population. Am J Public Health.

[29] Allanson ER, Schmeler KM (2021). Cervical cancer prevention in low- and middle-income countries. Clin Obstet Gynecol.

